# The Work, Economic, and Remittance Stress and Distress of the COVID-19 Pandemic Containment Policies: The Case of Venezuelan Migrants in Argentina and Chile

**DOI:** 10.3390/ijerph20043569

**Published:** 2023-02-17

**Authors:** Deisy Del Real, Felipe Crowhurst-Pons, Lizeth Olave

**Affiliations:** 1 Sociology Department, University of Southern California, Los Angeles, CA 90089, USA; 2 Departamento de Ciencia Política y Políticas Públicas, Universidad del Desarrollo, Santiago 7610315, Chile; 3 Escuela Interdisciplinaria de Estudios Sociales, Universidad Nacional de San Martín, San Martín 1650, Argentina

**Keywords:** Venezuelan migration, social stress process, remittance stressor, mental health, psychological well-being, health disparities, COVID-19 containment policies

## Abstract

According to the social stress process model, global crises are macro-level stressors that generate physiological stress and psychological distress. However, existing research has not identified immigrants’ COVID-19 containment policy stressors or examined the social stress of sending remittances amid crises. Drawing on in-depth longitudinal interviews with 46 Venezuelan immigrants—half before and half during the pandemic—in Chile and Argentina, we identified the COVID-19 containment policies’ stressors. We focused on Venezuelan immigrants because they constitute one of the largest internationally displaced populations, with most migrating within South America. We found that the governmental COVID-19 containment measures in both countries generated four stressors: employment loss, income loss, devaluation of employment status, and inability to send needed remittances. Moreover, sending remittances helped some migrants cope with concerns about loved ones in Venezuela. However, sending remittances became a social stressor when immigrants struggled to simultaneously sustain their livelihoods and send financial support to relatives experiencing hardships in Venezuela. For some immigrants, these adversities generated other stressors (e.g., housing instability) and symptoms of anxiety and depression. Broadly, for immigrants, the stressors of global crises transcend international borders and generate high stress, which strains their psychological well-being.

## 1. Introduction

Starting in March 2020, governments worldwide closed their borders and imposed social distancing restrictions to contain the COVID-19 pandemic, which resulted in economic downturns, business closures, and job loss [1,2,3]. According to the sociological social stress process model, the governmental COVID-19 containment responses likely generated a wide range of stressors that inflict stress and psychological distress on immigrants. Previous research has shown that macro-level changes that systemically impact societies, such as economic recessions, generate a wide range of stressors (i.e., unemployment) that can bring on secondary stressors (i.e., financial strain and identity threat) [4,5,6,7,8,9]. These multiple stressors heighten individuals’ physiological stress, drain their coping capacities (e.g., social support, self-regard, and sense of mastery), and strain their psychological well-being before turning into psychological distress (i.e., depression and anxiety) [4,5,6,7,8,9]. For example, global economic crises increase involuntary unemployment and underemployment stressors, which generate secondary stressors—associated with income loss and familial conflict—that deplete an individual’s sense of self-worth and generate psychological distress, such as depression [10,11,12,13]. Even people who keep their jobs during recessions can experience the stress and psychological distress of professional stagnation or demotion [8,14]. Conversely, individuals with sufficient coping resources (e.g., social support and a sense of self-mastery) can mitigate the stressors’ negative consequences and prevent them from experiencing psychological distress [4,5,6,7,8,9,14].

These work and economic stressors, stress, and distress dynamics more severely affect immigrants who are in structurally disadvantaged positions and already struggling with the psychological strain of underemployment, the loss of professional status they had in their country of origin [15], limited labor market opportunities, and precarious employment conditions [16,17,18,19,20,21,22]. The COVID-19 pandemic exacerbated these conditions, as immigrants experienced high psychological distress in general [23], or due to being in debt [24], a perceived threat of financial hardship [25], and when their employers cut their working hours [26]. Immigrants can draw on interpersonal support and other coping mechanisms to mitigate the negative consequences of these stressors and protect their mental health [21].

Although insightful, research on the COVID-19 pandemic’s impact on immigrants’ stress and psychological distress is nascent and does not fully specify the stressors of governmental efforts to contain this sanitary crisis. Most importantly, the social stress and migrant psychological well-being literature focus on the country of destination without considering the social stress of sending remittances to loved ones who did not migrate. An analysis of the social stress of sending remittances—or immigrants’ financial support to relatives in the country of origin—is needed because macro-level crises, such as the COVID-19 pandemic, worsen economic conditions in both countries [3]. Moreover, immigrants often move to other countries to work and send monetary support, or remittances, to loved ones in their country of origin [27,28,29,30,31,32,33,34,35,36]. Before the pandemic, many non-migrant people depended on remittances to sustain their livelihoods [27,28,29,30,31,32,33,34,35,36]. During the COVID-19 pandemic, many immigrants experienced work instability and income loss in their destination countries [3]. Simultaneously, their non-migrant relatives’ economic situation further deteriorated [3], increasing their need for financial support. Thus, remittances are likely a source of stress for immigrants unable to send the desired financial support to non-migrant loved ones. Alternatively, it is also possible that immigrants who can send the needed remittances experience relief. This predicament poses the question: How do global and multifaceted crises, such as the COVID-19 pandemic, generate stressors, stress, and psychological distress for immigrants seeking to sustain their livelihoods while sending remittances?

The present study provides a comparative and longitudinal analysis of how governmental COVID-19 containment policies impacted Venezuelan immigrants’ stress and distress processes in Chile and Argentina. We compare stressor experiences across two destination countries before and during the COVID-19 pandemic. Moreover, we consider stressors that affect immigrants due to changes in the destination countries and their ability to send remittances to loved ones in the country of origin. We conducted in-depth longitudinal interviews with 46 Venezuelan immigrants in these two countries—we interviewed the same migrants before and during the pandemic. We refer to our interviewees as immigrants because they did not apply for refuge or asylum; however, we do not challenge their right to claim such statuses [37,38]. We focus on Venezuelan migrants because more than 7.1 million have migrated to escape widespread human rights violations, crime, hyperinflation, scarcity of food and essential goods, and a deteriorated healthcare system [38,39]. Chile and Argentina are among their main destinations [39].

Findings show that governmental COVID-19 containment policies generated four stressors among some migrants: job loss and underemployment, income loss, employment status devaluation, and inability to send the needed remittances. In the country of destination, the work stressors brought on secondary stressors such as housing instability and anticipatory stress. These stressors were the source of self-reported physiological stress and, in several cases, generated symptoms of depression or anxiety. Moreover, sending remittances to the country of origin was a coping mechanism and a social stressor. When immigrants could send financial support, this appeased their concerns for loved ones in Venezuela. However, sending remittances became a stressor for Venezuelan immigrants who experienced adverse employment and household income changes. These immigrants endured stress and, in some cases, symptoms of depression and anxiety when unable to send sufficient financial support to loved ones struggling to sustain their livelihoods in Venezuela. Thus, we argue that immigrants’ social stressors accumulate and proliferate across international borders during global crises.

In the following sections, we review the stress process research and situate the case of Venezuelan immigrants in Chile and Argentina within larger macro-changes. Then, we review our methods and present and discuss the implications of the findings.

## 2. Social Stress Process Model

According to the social stress process model, it is reasonable to expect that governmental efforts to contain the spread of COVID-19 generated many stressors that undermine immigrants’ psychological well-being. The social stress process model posits that various socioeconomic and political contexts generate social stressors [4,5,6,7,8,9]. Stressors threaten an individual’s “stability of identity, role occupancy, social and network locations, or physical well-being” [14] (p. 300). Various social stressors exist, which manifest as discrete life events (i.e., job loss), daily hassles, non-events—desired events that should have but do not occur—or chronic, continuous situations without a clear end that gradually wear out an individual’s coping resources [14,40,41]. For example, anticipatory stressors are concerns about future hardships without an end in sight [7]. Exposure to one stressor can bring exposure to other secondary stressors [6,9]. For example, unemployment stress generates income loss and familial conflict stressors [6,9]. Stressors do not necessarily follow a particular order. For instance, familial conflict can generate the stress of job loss due to poor performance. As stressors accumulate and compound, stress proliferates and drains an individual’s coping capacities (i.e., self-regard, support from relationships, and sense of mastery) before generating psychological distress (i.e., anxiety and depression) [6,9]. Psychological distress is a “maladaptive response pattern in the presence of stress, such as anxiety, [and] depression” [14] (p. 300). 

Crises and large-scale societal changes generate multiple work and economic stressors that strain people’s psychological well-being [4,5,6,7,9]. For example, large-scale crises cause economic downturns that increase unemployment, underemployment, and stagnate job advancement [4,5,6,7,9]. Even in non-crises times, involuntary unemployment is a source of psychological distress because it negatively impacts people’s financial resources and identity [10,11,12,13]. If involuntarily unemployed people quickly find a new job, they are less likely to experience psychological distress [42]. However, job loss produces high stress and distress among people who struggle to find new employment [15,42] or find new jobs with lower pay, insecurity, or lower status than the ones they lost [8]. Moreover, underemployed workers experience distress [43,44]. Alternatively, individuals’ coping capacities can alleviate stress and prevent distress [6,9].

Before the COVID-19 pandemic, the stress of precarious jobs, involuntary unemployment, underemployment, and professional stagnation was more prevalent and severe for immigrants. Immigrants experience more work stress than citizens because they are more likely to have precarious and nonstandard bad jobs with high risk and security [8,16,17,18,20,21,22]. Specifically, immigrants who have undocumented or liminal legal status, are racialized, and have low educational attainment with fewer employment opportunities, forcing them to endure the stress and distress of bad working conditions and daily discrimination from coworkers and employers [21,22,25]. Even highly educated immigrants experience the stress of underemployment or a loss of professional status in the destination country [15]. Though, immigrants with more coping resources (e.g., robust social supports, lawful permanent residency, and a sense of self-mastery) can better cope with these stressors and prevent psychological distress [21].

The pandemic worsened the unstable work position of immigrants. The COVID-19 governmental containment policies disrupted labor markets and were likely a source of stress and distress for immigrants who saw their employment and economic stability uprooted. Research on aggregates of immigrant populations in wealthy and highly industrialized countries shows that the COVID-19 pandemic generated work stress and distress for immigrants due to the perceived threat of financial hardship [25] or the loss of working hours without protection [26]. An aggregate study based on a non-representative sample of 1008 Venezuelans in Chile found that 72% of respondents were sad and depressed due to the COVID-19 pandemic [23]. Venezuelan immigrants who felt unprepared to face the pandemic had higher anxiety and depression rates [23]. Although insightful, the pandemic generated global crises, and more research is needed to identify the COVID-19 containment policies’ sources of stress and distress in immigrants’ countries of origin and destination.

Specifically, this study examines the social stress of sending remittances, which likely was exacerbated by the pandemic. During the COVID-19 pandemic’s economic crises, immigrants and their non-migrant relatives simultaneously experience job instability and economic hardship [1,2,3]. Nonetheless, immigrants continued sending remittances to loved ones [36]. For example, at the onset of the pandemic, remittance flows to Latin America increased by 7.1 percent from 2019 to 2020 [36] (p. 1). The recipients of remittances often depend on this monetary support to sustain their livelihoods [3,35]. Hence, it is possible that sending remittances may have provided some solace for immigrants who were worried about loved ones in their country of origin. Conversely, sending remittances can become a social stressor for immigrants who struggle to support their and their non-migrant relatives’ livelihoods. Therefore, this study identifies the COVID-19 pandemic containment policies’ sources of stress and psychological distress for Venezuelan immigrants in their destination countries while considering their financial obligations abroad.

## 3. COVID-19 Containment Policies and Venezuelan Immigrants in Argentina and Chile

Venezuelan immigrants and refugees constitute one of the largest international displaced populations in the world [38,39]. The death of Venezuelan President Hugo Chávez (1999–2013) and the contested election of President Nicolás Maduro (2013–present) have generated mass emigration [37,38]. As of November 2022, more than 7.1 million Venezuelans have fled the country [39] due to widespread human rights violations, crime, and shortages of food, essential goods, and public healthcare resources [38]. More than 80% of Venezuelan migrants have moved to other South American countries [39]. Chile and Argentina are among the top six destination countries for Venezuelan immigrants and refugees [39]. According to data from December 2020, approximately half a million Venezuelan migrants resided in Chile [39]. Figures from June 2022 reveal that about 171,050 Venezuelan migrants resided in Argentina [39]. These estimates are based on immigrants who apply for lawful residency, undercounting the undocumented population.

The COVID-19 pandemic and governmental efforts to contain contagion deteriorated the already fragile economies of Latin American countries [1,2], which we can reasonably expect to have generated various stressors for Venezuelan immigrants. The economies and labor markets in Latin American countries were among the worst impacted by the pandemic, largely because half of the workforce, on average, is in the informal labor market [1,2,45]. Informal jobs are unproductive and highly unstable and do not contribute to workers’ social security or provide them benefits, such as health insurance, social security, and severance payments [45,46]. Thus, in 2020, Latin American and Caribbean countries experienced an approximate 21% decrease in hours worked, the biggest lost worldwide [2] (p. 12). Global hours lost was 12% [2]. 

Argentina and Chile experienced similar economic downturns and have high job informality rates [1]. Between 2019 and 2020, Argentina’s gross domestic product (GDP) decreased from 524,820 to 452,819 million dollars while Chile’s decreased from 297,572 to 279,385 million dollars [1] (p. 236). During this same timeframe, unemployment rates increased in Argentina from 9.8 to 11.5 and in Chile from 7.2 to 10.8 [1] (p. 256) (CEPAL estimate focuses on Argentina’s urban areas). Informal workers in both countries were the hardest hit; they lost jobs or stopped working due to the pandemic containment measures [1] (p. 81). 

Venezuelan immigrants were particularly vulnerable to these macro-economic changes [3]. Most Venezuelan immigrants work in the informal labor market or in service industries that were negatively impacted by social distancing measures [36,46,47,48]. Not surprisingly, a survey found that 24% (of 416) Venezuelans immigrants throughout the Americas lost income and the ability to buy food due to job loss because of the COVID-19 containment policies [3].

Despite the adverse economic changes of the COVID-19 pandemic, most immigrants did not benefit from the Chilean or Argentine governments’ monetary aid programs [49,50,51,52]. Specifically, most of the Chilean government’s monetary transfers disqualified informal workers [53]. These programs included paid unemployment benefits (“Ley de Protección al Empleo”), retirement withdrawals (“Retiro del 10% de la AFP”), family emergency income (“Ingreso Familiar de Emergencia”), nonrefundable money transfers for low-income and middle-income families (“Bonos COVID-19” and “Bono Clase Media”), and credit for middle-income families (“Préstamo Clase Media”) [53]. To support formal workers, the Argentine government increased unemployment benefits and subsidized their salaries (via “Programa de Asistencia de Emergencia al Trabajo y la Producción”) [48]. Moreover, the Argentine government issued Emergency Family Income (Ingreso Familiar de Emergencia, IFE) to provide monetary support to informal, domestic, self-employed, and other unemployed workers during the pandemic [48]. Finally, the Argentine government increased financial support to existing programs that helped vulnerable populations (i.e., retirees) [48]. 

Nonetheless, immigrants and informal workers had limited access to public COVID-19 assistance [45,46,49,51] because government officials could not reach these workers [46], and the programs had high requirements. In Chile, the pandemic aid programs required a valid RUT [53], which disqualified undocumented immigrants, those with expired RUTs [51,52], or those who were processing their lawful residency status and did not have RUTs. Similarly, in Argentina, immigrants struggled to access the government’s COVID-19 aid because they had not finalized their legal residency applications [50]. Legal residency application procedures were delayed during the digitalization process in both countries [50,54]. Moreover, many immigrants did not meet the Argentine IFE programs’ two years of residency requirement or struggled to prove they had lived in the country that long [50]. For example, a national non-representative sample of 3188 immigrants in Argentina between October and November 2020 found that only 20% of immigrants had accessed the IFE [49] (p. 8). Among them, Venezuelan immigrants had the lowest assistance rates; only 3% had applied and received the IFE benefit [49] (p. 9).

Despite the pandemic’s hardships and limited access to governmental support, Venezuelan immigrants needed to continue sending remittances to non-migrant loved ones in Venezuela who depended on this money to sustain their livelihoods [2,35]. A study found that the vast majority of the 2231 adult Venezuelan migrants surveyed sent remittances before and during the pandemic to cover the living expenses, healthcare costs, and debt of their parents, siblings, aunts, and uncles who stayed in the country of origin [35]. The same study found that for 81% of people in Venezuela who received remittances, this monetary support constituted their main or one of their main sources of income [35].

## 4. Data and Methods

To examine how the COVID-19 containment policies impacted Venezuelan immigrants’ stress and psychological well-being, we drew on semi-structured and in-depth interviews with Venezuelan migrants in the Metropolitan Region, Chile, and Buenos Aires, Argentina—or the urban center where migrants tend to be concentrated [37]. We conducted in-depth interviews with 46 Venezuelan migrants between 2018 and 2019 and follow-up-interviews with the same immigrants after June 2020. The first interviews are part of a larger study that began in 2018. When the COVID-19 pandemic started, we conducted follow-up interviews with the same 46 Venezuelan migrants to assess the impact of the crises. Specifically, we conducted 42 interviews in Argentina and 50 interviews in Chile—for each country, half were before and half after the pandemic’s start. To ensure that national origin does not explain the different outcomes, we only interviewed Venezuelan immigrants who were 18 years or older, had lived in Argentina or Chile for more than 90 days, and intended to stay. We did not interview tourists or those transiting the country. 

We used a snowball sampling technique to recruit participants and asked respondents and community partners to refer us to new interviewees. We collaborated with four key respondents from two migrant-serving non-governmental organizations and two Venezuelan associations to recruit hard-to-reach respondents, increase sample variability, and verify preliminary findings. Before the pandemic, in-person interviews were conducted in locations where interviewees felt comfortable, such as cafes, parks, and their homes. During the pandemic, we conducted interviews via phone, Skype, WhatsApp, and Zoom to abide by social distancing measures.

As seen in Table 1, Venezuelan interviewees in both countries had similar demographic characteristics. The average age of interviewees was 40 in Argentina and 35 in Chile. We had gender parity in Argentina and interviewed more men in Chile because migrants are mainly males (Gandini et al., 2019). None of the interviewees identified as non-binary. In both countries, most interviewees had at least some college education or higher and came from middle to middle-high socioeconomic backgrounds in Venezuela. At the time of the first interview, the monthly household income of interviewees in Chile was, on average, USD 1352—the range was from USD 318 to 2541. Five interviewees had a household income below the poverty line in Chile. In comparison, the average monthly household income for Venezuelan interviewees in Argentina was USD 802, ranging from USD 122 to USD 1951. Only two interviewees had a household income below the poverty line in Argentina. Finally, interviewees had resided an average of two years in Chile and an average of five years in Argentina. Overall, the interviewees in both countries are comparable. 

Two in-depth, semi-structured interview protocols were developed and modified throughout the interview processes as new themes emerged. To measure the impacts of the COVID-19 containment policies, we conducted two sets of in-depth interviews with the same immigrants. The first interviews, between 2018 and 2019, are part of a larger study. The first questions focused on Venezuelan migrants’ employment, economic stability, household income, ability to send remittances, social ties, healthcare access, and psychological well-being. Similar questions were used in the 2020 follow-up interviews. In addition, we asked migrants what they considered the most challenging aspect of the pandemic, what they worried about daily, and whether the COVID-19 containment measures impacted their employment, economic stability, household income, and ability to send remittances to their loved ones. Answers to the first interviews provided a threshold to help us identify how the pandemic changed immigrants’ socioeconomic situations and remittance patterns. The first interviews strengthened our analysis as many interviewees did not remember aspects of their lives before the pandemic, such as their monthly household income.

Further, to identify symptoms of psychological distress (in terms of depression and anxiety), we used questions from the World Health Organization (WHO) World Mental Health (WMH) survey initiative. The WHM is a version of the WHO Composite International Diagnostic Interview (CIDI) for cross-national studies. Thus, if interviewees experienced difficult changes due to the COVID-19 containment policy, such as sending fewer remittances, we asked them to provide examples and probed how these experiences impacted their ability to go about their daily routines, emotions, and views of the future. These probes helped us identify whether the stressors brought on by COVID-19 containment policies generated symptoms of psychological distress. Finally, to measure how immigrants coped with stressors, we asked respondents to describe their social, emotional, and instrumental supports and how they soothed themselves during difficult times.

We analyzed interviews using HyperResearch software and followed an inductive process. We coded changes brought on by the COVID-19 containment policies as stressors when interviewees considered them strenuous or difficult to adjust to or endure [21]. The pandemic changed immigrants’ lives in non-stressful ways (e.g., some were promoted at work). We conducted frequency counts of all changes but did not consider these latter changes as stressors. Finally, we coded for symptoms of depression (including persistent sadness, discouragement, hopelessness, lack of interest in activities they usually enjoyed, or changes in eating and sleeping patterns) and anxiety (or persistent or excessive worry and fear about the future). Our measurement of psychological distress does not fully implement the WHO WMH questionnaire; instead, we identified qualitative evidence of anxiety and depression symptoms. Finally, we conducted frequency counts of how many interviewees showed signs of psychological distress (or anxiety or depression symptoms) due to the COVID-19 containment policy stressors. The following section delves deeper into these COVID-19 stressors for immigrants.

## 5. Results

The COVID-19 pandemic’s social distancing measures have exacerbated the economic hardships and work stress of some Venezuelan migrants in Argentina and Chile. Most interviewees fled Venezuela’s crumbling economy and were adjusting to the labor market in their destination countries when the pandemic shook their lives. Our analysis comparing migrants’ situation before and soon after the COVID-19 social distancing measures went into place reveals four main stressors brought on by the pandemic. As seen in Table 2, these stressors include employment loss and underemployment, decreased household income, decreased remittances sent to relatives in Venezuela, and a devaluation of employment status and prestige. Among 28 of the 46 interviewees—13 in Argentina and 15 in Chile—the COVID-19 containment policy stressors accumulated, reduced immigrants’ coping resources, and generated symptoms of anxiety or depression (or psychological distress).

### 5.1. Unemployment, Underemployment, and Income Loss Stressors

Specifically, employment loss and underemployment due to the COVID-19 pandemic include dismissals, furloughs, wage cuts, quitting one’s job to avoid contagion, and inability to work full-time or continue small business enterprises (e.g., street vending). Employment loss and underemployment were sources of stress for a third of the Venezuelans we interviewed in both countries (see Table 2). The stress of forced unemployment and underemployment often brought on the secondary stress of a decrease in Venezuelan immigrants’ household income. Specifically, 62% of the 21 Venezuelans we interviewed in Argentina, and 44% of the 25 interviewees in Chile experienced decreased household income (see Table 2). Often, immigrants endured the stress of these adverse employment and income changes without government aid.

For example, Antonio migrated to Chile seeking employment opportunities. In Chile, he secured jobs in fast-food restaurants as a manager. He left his first job because he disliked the work environment and quickly found another job as a manager of an international fast-food chain store where he was happy. However, Antonio explained during our interview that the pandemic’s social distancing measures caused him to lose his new job:

*I had a three-month contract that was supposed to become an indefinite one. However, the pandemic started, and then they [the employer] gathered us and suspended or terminated our contracts.*

Before the pandemic, Antonio had a good job and economic stability; he was earning 1,000,000 Chilean pesos a month (about US$1400), which was 2.4 times over the monthly poverty line for a household of four in Chile (see Table 1). However, the employer lost profits because they had to abide closely by the COVID-19 containment policies. To mitigate the loss, the employer fired and laid off several employees who had not completed their 90-day probationary periods. Since Antonio lost a job in the formal labor market and was a lawful permanent resident, he applied and received government unemployment aid of 233,000 Chilean pesos for three months (or about US$326). Moreover, at the time of the interview, he was figuring out how to apply for a government rent subsidy. After his three-month unemployment aid, he survived by selling street food with his wife. Antonio’s monthly household income plummeted from 1,000,000 to 150,000 Chilean pesos (or about US$1400 to US$210), which was below the poverty line. 

Antonio’s sudden loss of formal employment and income became a source of high stress and anxiety. During our interview, he explained:

*We are living in uncertainty. Without employment, without producing, obviously, I am worried. I’ve been really worried for months because I am running out of money. If I get kicked out of the apartment because I can’t pay, where am I going to live? Where am I going to take my family? So, when they take away your [job] stability, your shelter is also at risk. It would be like starting from scratch. I will go back to how I was when I arrived because when I arrived [to Chile], I was at a friend’s house, on an inflatable mattress, sharing a bed, and in a small space.*

Losing his job was a major event stressor for Antonio that created secondary stressors and generated psychological distress. His loss of income was putting his housing situation at risk; he feared his family would be evicted one day. Moreover, Antonio felt the daunting stress of losing what he had gained; his insertion into the Chilean labor market severely regressed. Losing these successes was a source of stress because Antonio felt he had to start from scratch in Chile. Moreover, Antonio had anticipatory stress because he did not know what new containment policies the government would enact or how they would affect his ability to find new employment in the foreseeable future. Antonio struggled to find a locus of control. He did not know when or how to resolve his problems because the pandemic and the governments’ containment policies were highly unpredictable. The stress proliferation destabilized Antonio’s psychological well-being as he showed signs of excessive and persistent concern and fear of the future or anxiety.

Similarly, Sonia was a lawyer in Venezuela and moved with her husband and two children to Chile to escape their country’s ongoing crises but experienced high work and economic stress and anxiety in Chile during the pandemic. She had been working as an informal housekeeper in Chile when the government imposed social distancing measures to contain the COVID-19 pandemic. In response, Sonia’s employer:

*Told me that I could no longer go [to work]. … They talked about suspending the working relationship. But, since I did not have a contract or anything that legally bound us, I did not receive any payment, neither the full income nor the half, so that has obviously forced me to look for another source of income.*

Sonia’s informal employment left her vulnerable; her employer fired her without paying her all her pending wages or giving her any severance. Furthermore, Sonia’s husband was self-employed and could not continue catering events during the pandemic. They did not qualify for government aid because they worked informally. As Sonia’s situation illustrates, immigrants in the informal labor market often face adverse employment and income changes without government assistance.

To survive, Sonia and her older daughter found jobs as part-time seamstresses at a factory, which only paid them about 900,000 Chilean pesos a month (about USD 140). Sonia and her daughter’s part-time factory jobs became the family’s primary source of income to pay for essential goods, services, and rent. At the onset of the pandemic, the family’s monthly household income decreased from 400,000 to 90,000 Chilean pesos a month (from USD 560 to USD140). To cope, the family cut phone services and bought less food. They did not apply for government aid because they were still processing their legal residency applications and could not meet the requirements (e.g., providing a RUT, or Chilean identification card). Sonia and her husband received food from their younger child’s schools (a non-governmental source of assistance) and verbally agreed with the landlord to pay the rent in installments while struggling economically. However, they had no legal protection and remained vulnerable to the landlord’s ability and willingness to continue the informal arrangement. 

Since the family’s monthly expenses vastly surpassed their low income, their sense of economic security vanished. Together, the family’s job losses, underemployment, and income loss were a source of high stress that brought on secondary stressors. Sonia explained:

*The most challenging thing for me is the emotional part, the fear of being unable to meet the obligations, especially the rent. And suddenly, the landlord will say that we must give up the apartment and that my children, husband, and I will be forced into the streets. … Although I thank God we have come across people with good hearts, it is not easy for anyone to help anybody else in this situation. So, it is really my great fear, you understand, because even though they have opened many shelters, we are not used to that situation. … Beyond that fear, I feel pain for my children. It is hard for my husband and I to expose our children to this vulnerability.*

Sonia struggled to deal with the emotional and psychological burden of her family’s vulnerable economic situation. Despite seeking solutions to mitigate her situation, Sonia lived with the stress of meeting living expenses with a lower household income, the anticipatory stress of becoming homeless, and the pain of seeing her children suffer. The stressors proliferated into distress, and Sonia was experiencing anxiety as she felt constantly worried and fearful about her family’s future. 

Similar trends unfolded in Argentina, where several Venezuelan migrants experienced the stress and distress of unemployment, under-employment, and income loss due to the pandemic containment policies. For instance, Victor commuted into Buenos Aires to sell avocados on the streets before the pandemic. However, as part of the COVID-19 social distancing measures, the government banned street vending and required permits for people to enter Buenos Aires city (the capital). These measures were devastating for Victor, as he explained:

*Because you cannot sell, you cannot go out to sell on the street, if you are from the [Buenos Aires] province, you cannot enter the capital [of Buenos Aires]. So, they sometimes take measures that… either they are in a single alignment, or they are not. … When the quarantine began in mid-March, they asked me for it (permission to circulate).*

Victor was undocumented and did not have the Argentine identification card number (DNI) or proof of formal employment, which he needed to get a circulation permit to travel from his home in La Matanza (in greater Buenos Aires) to the capital of Buenos Aires. To sell goods, he traveled to the capital without a permit and faced constant police scrutiny. Unable to work or circulate the city, Victor explained how his emotional health was negatively affected:

*Work is a form of distraction; imagine… you are alone. Are you going to be locked up in a house all day? Imagine… if you have a TV, you have internet, but if you had none of that? … The mind begins to wonder. If I die, what is going to happen… to my family? … It is a difficult situation, we hope to get out of it unscathed because this year, there is practically no existence. In 2020, there is nothing.*

Victor’s situation shows that for some migrants, job loss is a source of stress and depression because work becomes the activity that gives meaning to their existence, especially those who have left their entire family in their country of origin. When he lost his job, Victor lost the income he needed for survival and his sense of purpose for migrating, living away from family, and enduring daily deprivations in Argentina. Further, he could not afford television or internet services, so he did not have distractions or an easy way to communicate with his family back in Venezuela. Work had been Victor’s way of earning a living, passing his time, and fulfilling a purpose. Unable to sell avocados, he struggled to cope with the multiple stressors of figuring out how to survive alone, away from the support and comfort of his loved ones. Despite his dire need for help, Victor’s informal employment and unresolved lawful residency status made him ineligible for government aid. 

Indeed, most interviewees who experienced adverse employment and income changes during the pandemic did not receive governmental COVID-19 aid. Only fourteen of the forty six interviewees in both countries received monetary assistance from the government to counter the economic hardships of the COVID-19 pandemic. They all worked in the formal labor market and had the required identification documents. The public aid they received included: unemployment benefits, direct monetary transfers, subsidized salaries to prevent their layoffs, and subsidized housing. In contrast, none of the fifteen Venezuelan immigrants in the informal sectors received public COVID-19 monetary transfers. Informal migrant workers’ occupations, limited time of residency, or lack of finalized lawful residency applications obstructed their access to public aid. 

### 5.2. Remittances: A Coping Strategy and a Social Stressor

Despite their hardships, Venezuelan immigrants wanted to support their relatives in Venezuela who faced the multiple crises afflicting their country of origin. The economic crises of the COVID-19 pandemic and containment policies also impacted the relatives of interviewees who remained in the crises-induced Venezuela. The situation in Venezuela became a source of anticipatory stress for many Venezuelan interviewees as they worried about how much conditions would deteriorate with the pandemic and whether their parents and other relatives could access healthcare and other essential goods. Venezuelans who had migrated for a better life felt compelled to support their loved ones by sending them money (or remittances).

In terms of the stress process, remittances served two functions. For some Venezuelan migrants who could send as much as they wished, sending money became a coping mechanism to appease concerns for their loved ones. However, for others who could not send as much as they wanted, their inability to provide financial support became a source of secondary stress. 

Specifically, some Venezuelan interviewees could send the same or more remittances to loved ones during the pandemic (including 11 of the 25 interviewees in Chile and 6 of the 21 respondents in Argentina). Their financial support helped appease some of their concerns. For example, Sandro, who worked as a head of management in a company, doubled his remittances to his parents because he was afraid that the pandemic would hit them hard. He explained:

*Look, in my case, I have increased [the remittances] a little because I understand that the situation in Venezuela is very critical, so I am more worried because my parents are at risk of the coronavirus, and so I worry more. … Moreover, Venezuela is going through a very delicate economic crisis, and inflation is hyperinflation, so if you add that to the fact that people cannot go out because they are in quarantine. They do not even have gasoline to move around the country; then, it is difficult. And right now, if we can help, we will continue to do so.*

Sandro had the economic resources to increase his remittances to Venezuela. During the 2020 interview, he earned 1,400,000 Chilean pesos (about USD 1961) monthly. 

Similarly, Gustavo (a community program developer in Chile) started sending more money to his parents during the pandemic. Gustavo earned a high income in Chile, and his remittance increase only resulted in minor adjustments to his household budget. It did not impact his family’s sense of economic stability. In this sense, increasing the money he sent to his relatives in Venezuela was a coping mechanism. During our interview, he said:

*I continue to send remittances regularly. In fact, I have increased the money I send because of the urgency. I was in Venezuela in February [2020]. … It was great being there but very concerning. The most difficult part is the uncertainty, not knowing when it will end. … There have been news reports that Venezuela now has no gasoline and is buying gasoline from Iran. They are also in quarantine, and the real numbers of [people with coronavirus] are unknown, so the problem has worsened severely, and my family requires more money.*

Gustavo also experienced anticipatory stress for his family situation in Venezuela. For them, the pandemic reinforced the political and economic struggles that Venezuela has been experiencing since Maduro’s regime began in 2013. At the time of the interview, Gustavo and his partner earned 1,200,000 Chilean pesos a month (or about USD 1681). To appease their worries and thanks to their high income, they sent more money to their families in Venezuela when the pandemic hit. As such, their remittances served as a coping strategy to mitigate their anticipatory stress. 

However, for other immigrants, remittances were a source of stress because they were unable to provide as much economic support as they wanted to their loved ones in Venezuela during the pandemic. Approximately half of the 21 interviewees in Argentina and more than a third of the 25 interviewees in Chile sent fewer remittances when the pandemic started due to employment loss, wage cuts, and other unforeseen expenses. For these Venezuelan migrants, the anticipatory stress and concerns about their non-migrant loved ones were exacerbated by their inability to help them. 

For example, at the start of the pandemic, Patricia lost her jobs as a part-time teacher and a caregiver to an older woman. Her positions were informal, so she did not receive any severance. She lived with her adult daughter and son-in-law in Santiago, Chile; her son-in-law was laid-off and receiving severance. Her daughter could continue working from home and helped support the three of them by taking an extra job delivering pizzas. Moreover, Patricia began selling Venezuelan street food to earn income to support her household in Chile and her father in Venezuela. She was worried about her father and wanted to send him more money, but she was economically strained and trying to find ways to limit her expenses in Chile. During the interview, she explained:

*We help my dad, who is in Venezuela. … I left my pension to him; I do not collect my pension; he collects it over there. …. We used to send 30,000 [Chilean pesos, clp], and 40,000 [clp] monthly, but now we cannot do it. We send 10,000 [clp], we send 15,000 [clp], whatever we can because our situation is challenging now.*

Due to the COVID-19 containment policies (e.g., school closures), Patricia was dealing with multiple stressors: job loss, income loss, concerns about her father’s livelihood, and the inability to send him more money. To lessen the stress, she was “practicing my meditation techniques, prayers, and yoga for many years, … which has allowed me to take things in a very different way.” Nonetheless, Patricia’s coping strategies only helped appease some of the stress of the situation. Still, the underlying problem remained unresolved. Despite having ongoing anticipatory anxiety about her father’s well-being, Patricia had to decrease her financial support and could not find a way to improve her economic circumstances.

The stressors surpassed the coping capacities of other interviewees, such as María, who lived in Buenos Aires, Argentina, and stopped working as a taxi driver to avoid becoming infected with COVID-19. Although her husband continued to work, she struggled to find new and safer employment. Additionally, rising inflation in Argentina increased the cost of living, which made it difficult for María to continue sending remittances to loved ones in Venezuela. Decreasing remittances was a source of stress and anxiety (or persistent concern and fear):

*For me, the most difficult thing is knowing that my family in Venezuela is going through a hard time… it consumes most of my thoughts and worries. … That issue with the remittances is one of the most difficult issues for me, honestly.… We send remittances through the black-market dollars, so with each devaluation [of the Argentine peso] and each rise in the blue dollar is horrible! … We used to send remittances to four people; now we can only send them to two. And we send the same amount! In Venezuela, the conditions have worsened. … So, damn, we decided to send to the people in the worst situations. … These two people live from remittances, which is not enough, but we cannot send them more, and we can’t stop sending either.*

María has loved ones in Venezuela who depend entirely on remittances to survive, so her inability to find a new and safer job, the rising inflation in Argentina, and devaluing the money she sends caused her great stress and psychological distress. As María explained, Venezuelan migrants in Argentina often send remittances through the black market or using cryptocurrencies as official Argentine money-wiring services go through official Venezuelan currency markets, further devaluing their money. Nonetheless, the black remittances markets are also affected by macro inflation. 

As María highlights, the inflation in Argentina diminished the purchasing power of the money they sent—when her family sent the same amount, it translated into less. Thus, María constantly worried about how to continue sending money and felt distraught about having to stop sending monetary support to two of four relatives. For her, sending money to other loved ones did not bring her solace or appease her anticipatory fears that something terrible would happen to them in Venezuela. Instead, María lived with anxiety; she feared the day she could not send enough to support the livelihoods of relatives who depended on her to survive.

Similarly, Tatiana continued sending remittances to her elderly parents even though her employer cut her salary during the pandemic. Tatiana was a primary school teacher in Buenos Aires and lived with her Argentine partner and their one-year-old daughter. As a result of school and business closures during the pandemic, Tatiana’s and her partner’s salaries were cut by 10% and 30%. At the same time, her parents in Venezuela needed more financial support. Tatiana described the impact of these changes during the interview:

*Venezuela has so many problems that I don’t know if they have taken in the magnitude of this pandemic. … For example, those who sell vegetables from the State of Táchira do not arrive with the products to Caracas, so they have been costly. There are so many problems that this is one more problem for them. My dad is 80, and my mom is 73; they get along as best as they can. … My sister and I worry a lot, and we always tell them not to go out.*

Tatiana experienced anticipatory stress as she constantly worried about her elderly parents’ ability to access and pay for food and other essential goods as well as not getting COVID-19. For her, ceasing to send them money was not an option even when her household income decreased, yet the cost of food and other basic items increased due to inflation in Argentina. Tatiana explained how they coped:

*We had to tighten our belts. … Almost 50% of my salary goes to Caracas, and … support what we can support. There are food items that we do not buy now, we cut out foods. … And, well, we stopped saving because of that percentage that they cut from our salaries, and with what I am sending to Caracas, we cannot save. … I feel that food has gone up a lot [in Buenos Aires]… we started going to buy at fairs around here and asking chat groups where they bought things.*

Unable to change the macroeconomic strains in Venezuela or Argentina or earn more income, Tatiana and her partner continued sending remittances to her elderly parents in Venezuela and coped by doing things that were secondary stressors—ceasing to save money made her fear being unprepared for future emergencies (anticipatory stress) and having to continuously figure out how to cut food expenditures despite rising inflation (daily hassle stress).

### 5.3. Loss of Job Prestige and Status Stressors

Finally, adverse changes in the immigrants’ perceived status and prestige of their employment situation were prevalent; 57% of interviewees in Argentina and 36% of interviewees in Chile endured this stressor (see Table 2). For example, the pandemic containment policies destabilized Hugo’s high-income and stable job as an analyst at a telecommunications company in Chile. He explained:

*My boss had already given me the ok. I was going to be promoted, and all that was paralyzed. … My boss said that there is no hiring, and no promotions; they are talking about suspensions and dismissal, and I could be one more victim of that situation. … Emotionally, I feel a little stressed because I don’t know if tomorrow, they will fire me. I don’t know whether they will suspend me tomorrow. If that happens, getting a job in this complex coronavirus situation. To get a new job, I will have to look for other alternatives like doing Cornershop [a delivery app] or what do I know.*

The COVID-19 containment policies not only derailed Hugo’s promotion, but also increased the stress of losing an advancement in status and prestige as well as the anticipatory threat of losing his employment (one that had been stable before the pandemic). His anticipatory stress was becoming an excessive and generalized concern—or anxiety. Unlike many other interviews, Hugo’s stress was not due to a loss of his income. On the contrary, during the pandemic, he received an increase in income from 900,000 to 1,300,000 Chilean pesos. However, the company’s hiring and promotion freeze during the pandemic; the layoff threat became ongoing nonevent and anticipatory fear stressors that left him frustrated and apprehensive of the future. 

The pandemic containment policies also resulted in Jose Luis experiencing a loss in employment status and prestige; he went from working as a skilled artisan in Argentina to delivering food. During our interview, he explained:

*For me, the most complicated, difficult part of the COVID situation is not being able to go out to work. I could not keep my job because I worked in a carpentry shed. During the quarantine, they closed the company, and I lost my job; they didn’t call me back anymore. … I am working part-time using a delivery app.*

The loss of occupational status and prestige was the most challenging thing Jose had endured during the pandemic. For him, the source of stress did not have to do with a loss in income. On the contrary, he earned about the same monthly income using his bike to deliver food via an online application and continued paying for his family’s living expenses. The change in occupations produced ongoing stress because he lost the sense of pride in working in a job that allowed him to use the skills he had developed in Venezuela.

Similarly, before the pandemic, Marcos had landed his first big acting and dancing roles in Argentina, which he believed would propel his artistic career forward. However, during the pandemic, these projects were canceled or indefinitely postponed, generating psychological distress—or symptoms of depression such as hopelessness. Marcos described the sudden change:

*The most challenging part has been seeing how the [acting and dance] projects that I had, that I felt were going to be great steps that I would take in my life, collapsed in the blink of an eye. … I prefer to live from day to day because it seems that this situation has led us to walk into nothingness itself. … I avoid planning for anything in the future. At the beginning [of the pandemic], I did. I said next month I’m going to do a play, next month… and in the end, it ended up falling apart. We could not sustain it.*

Marcos had pinned his hopes on permanently changing his working life and dedicating himself to what he considers his passion—dancing and acting. However, as the filmmaking and dancing industries had to shut down to maintain social distancing measures, projects fell apart, and Marcos lost his big breaks. As the months passed, he saw his opportunities disappear. Marcos was showing signs of psychological distress in terms of depression. The loss of status and prestige Marcos had gained from the roles disappeared, leaving him discouraged and hopeless about the future.

### 5.4. Coping Strategies

Although legally entitled to public healthcare in both countries, none of the Venezuelan immigrants sought to access mental healthcare services to cope with these various stressors. Instead, they coped with the stressors of the pandemic by meditating, praying, exercising, spending time with loved ones, cutting money spent on shelter and essential goods, taking on extra jobs, and avoiding thinking about the future. 

The lack of mental healthcare utilization is surprising in Argentina because most Venezuelan immigrants knew about and felt comfortable navigating the country’s universal public healthcare system, which does not require immigrants to have legal status (Article 8 of Argentina’s Immigration Law N. 25,871, 2003). In contrast, most interviewees were wary about how to navigate Chile’s more complicated healthcare systems. Specifically, immigrants can access Chilean private and public healthcare free of charge if they have a life-threatening emergency (Chilean Ministry of Health 2019). For more comprehensive healthcare, immigrants with lawful residency and a RUT (the Chilean identification card) can sign-up for the public healthcare system (FONASA) or obtain insurance to access the private healthcare system (ISAPRES) [58]. Undocumented immigrants can apply for a provisional RUT to register and access public healthcare under FONASA’s section A (Chilean Ministry of Health, 2019). Despite the last option, most interviewees processing their lawful residency applications did not have or did not know they could get a provisional RUT to access public healthcare. 

Overall, governmental efforts to contain the COVID-19 pandemic destabilized the labor market insertion of most Venezuelan immigrants, generating multiple stressors from the countries of origin and destination that, at times, proliferated and caused distress. Generally, Venezuelan immigrants were left to fend for themselves; the vast majority did not qualify or were rejected from government aid programs meant to mitigate the hardships of the pandemic and did not access mental healthcare services. 

## 6. Discussion

We contend that, during global crises, immigrants’ social stressors transcend international borders and strain their psychological well-being. Specifically, findings show that governmental efforts to contain the COVID-19 pandemic generated four stressors: job loss, income loss, employment status devaluation, and inability to send the needed remittances. In Chile and Argentina, the adverse employment changes brought on secondary stressors such as anticipatory stress and housing instability. For several Venezuelan immigrants, the stressors and physiological stress in their destination countries drained their coping resources and generated symptoms of psychological distress (depression and anxiety). Moreover, some interviewees experienced sending remittances to their country of origin as a stressor. As many Venezuelan immigrants faced the stress of economic hardships in their countries of destination, they also endured the stress and, in some cases, psychological distress (anxiety) of not being able to send sufficient remittances to loved ones struggling to sustain their livelihoods in Venezuela. 

These findings contribute to the social stress process model in several ways. First, previous research on immigrant social stress and psychological well-being mainly focuses on stressors that emerge within the destination country, e.g., [8,17,18,19,20,21,22,25,26,59]. The present study advances existing research by identifying how sending remittances can become a source of social stress that compounds and proliferates other stressors in the destination country. Broadly, we contend that remittances can serve as a coping mechanism for concerned immigrants who can send monetary support to non-migrant loved ones. However, remittances become a source of chronic stress and psychological distress for immigrants unable to send the needed monetary support to relatives in the country of origin. 

Specifically, the present study shows that, during the pandemic, Venezuelan migrants endured anticipatory stress as they worried about how their non-migrant relatives would face the pandemic in a country already inflicted with multiple crises. Those who maintained their jobs or income levels felt a sense of relief when they could send the same or more remittances to relatives in Venezuela. However, as some Venezuelan immigrants lost income, jobs, and working hours in Chile and Argentina, the need to send money to relatives in Venezuela incremented. Several immigrants experienced stress, and some had symptoms of depression or anxiety when they could not send the needed financial support to their loved ones. Since non-migrants need monetary support regularly, when immigrants cannot send the needed financial assistance, remittances become a chronic social stressor. The ongoing social stress of sending remittances exacerbates the psychological hardships of immigrants’ unemployment, underemployment, and loss of income.

Moreover, this study sheds light on the COVID-19 social stressors of humanitarian immigrants (or internationally displaced persons) who reside in middle-income countries in the Global South. Existing social stress process research tends to focus on citizens and immigrants in highly industrialized and wealthy countries of the Global North, for reviews see: [9,60]. However, working conditions in the Global South tend to have more precarious labor markets for citizens and immigrants than in the Global North. For example, Latin American countries’ economies were among the most negatively impacted by the COVID-19 pandemic containment efforts because approximately half of workers were in the informal labor market and did not have social security protections when they faced underemployment and unemployment [1,2,45,46]. Our findings capture these patterns. Venezuelan interviewees who worked in the informal sector tended to experience work-, economic- and remittance-related stress and, in some cases, symptoms of psychological distress. Moreover, immigrants in the informal sector were less likely to qualify for government aid to counter the hardships of the sanitary and economic crises. 

Finally, this study contributes to the nascent but growing research on Venezuelan migrants’ psychological well-being. Previous studies found that Venezuelan immigrants experience psychological distress due to societal discrimination [61,62,63,64], fears about education and career status loss in the destination country [65], and the COVID-19 pandemic in general [23]. Moreover, there is evidence that Venezuelans in Peru experience stress when unable to send remittances during the pandemic [66]. The present research contributes to this literature by identifying the specific stressors of the COVID-19 pandemic containment policies, finding evidence that they produce some symptoms of distress, and specifying when social remittances serve as a coping mechanism or become a stressor. 

This study has some limitations; thus, future research can expand on this research. Specifically, this study does not examine differences in immigrants’ demographic characteristics or quantitative trends and only examines the immediate impact of the COVID-19 pandemic. Hence, future research can examine larger timeframes and compare differences by gender and socioeconomic background. Additionally, mixed method studies can include quantitative analyses to determine the timing, directionality, and impact of these immigrant-specific stressors, especially remittance stress. Moreover, scholars can further explore the psychological toll of remittances and how other dynamics and obligations in the country of origin (e.g., violence and businesses) impact immigrants’ stress and well-being in the destination country. In addition, studies can identify the stressors of people who depend on remittances and other support from loved ones who emigrated. Further, scholars can continue identifying and comparing the stressors that more severely or uniquely impact immigrants’ psychological well-being in various parts of the world. Finally, future research can identify what government aid programs helped immigrants withstand and thrive amid global crises. 

## 7. Conclusions

In conclusion, governmental efforts to contain the COVID-19 pandemic disrupted economies and strained immigrants’ psychological well-being without providing adequate support to endure these policies. Our study indicates that Venezuelan immigrants are more likely to struggle to access government aid and healthcare services when they work in the informal labor market, do not meet residency requirements, are undocumented, or have not finalized lawful residency applications. Policymakers and advocates are encouraged to expand immigrants’ access to safety net programs and mental healthcare services as well as make these resources available regardless of immigrants’ time of residence, type of employment (formal or informal), or lawful residency status.

## Figures and Tables

**Table 1:** Demographic characteristics of interviewees in Chile and Argentina (first interview).

Demographic Characteristics	Country of Destination	
	Chile	Argentina
Total interviews	25	21
Average Age and Range	40 (23 to 65)	35 (22 to 51)
Women	10	9
Men	15	11
Non-binary	0	0
Educational attainment		
High school or less	1	0
Some college or technical college degree	5	10
Bachelor’s degree or higher	19	11
Socioeconomic background in Venezuela *		
Middle-high to high	6	4
Middle	15	15
Low	4	2
Average monthly household income in destination country	USD 1352	USD 802
Range of monthly household incomes in destination country	USD 318 to 2541	USD 122 to 1951
Monthly poverty line for a household of four **	USD 572	USD 451
Number of interviewees living in households below the monthly poverty line	5	2
Average Time of Residence at Destination and Range	2 years (1 to 4 years)	5 years (8 months to 17 years)

* For more on how we coded SES background in Venezuela see [55] (p. 10). ** The estimates of the poverty line for a household of four refer to CLP 450,165 for 2020 in Chile [56], and AR$12,087 or US$451 for 2019 in Buenos Aires, Argentina [57].

**Table 2:** The stressors of the COVID-19 governmental containment policies for Venezuelan migrants in Argentina and Chile, Frequency (%).

Stressors Due to COVID-19 Pandemic Containment Policies	Country of Destination	
	Argentina (N = 21)	Chile (N = 25)
Employment Loss or Underemployment Stressor *		
Yes	8 (38%)	9 (36%)
No	13 (62%)	16 (64%)
Household Income Loss Stressor		
Yes (income decreased)	13 (62%)	11 (44%)
No (income the same)	6 (29%)	6 (24%)
No (income increased)	2 (10%)	8 (32%)
Sending Remittances Stressor		
Yes (sent less)	9 (43%)	8 (32%)
No (sent the same or more)	6 (29%)	11 (44%)
Not Applicable	6 (29%)	6 (24%)
Employment Devaluation in Prestige or Status Stressor		
Yes (worsened or nonevent)	12 (57%)	9 (36%)
No (no change)	6 (29%)	9 (36%)
No (improved)	3 (14%)	7 (28%)

* Employment loss includes being fired, being furloughed, or leaving a job to prevent contagion. One interviewee arrived during pandemic and had not found employment.

## Data Availability

Data is unavailable due to privacy and ethical restrictions.
